# Dose-dependent effects of small-molecule antagonists on the genomic landscape of androgen receptor binding

**DOI:** 10.1186/1471-2164-13-355

**Published:** 2012-07-31

**Authors:** Zhou Zhu, Manli Shi, Wenyue Hu, Heather Estrella, Jon Engebretsen, Tim Nichols, David Briere, Natilie Hosea, Gerrit Los, Paul A Rejto, Andrea Fanjul

**Affiliations:** 1 Oncology Research Unit, Pfizer Worldwide Research & Development, La Jolla Laboratories, San Diego, CA, 92121, USA; 2 Drug Safety Research & Development, Pfizer Worldwide Research & Development, La Jolla Laboratories, San Diego, CA, 92121, USA; 3 Pharmacokinetics, Dynamics & Metabolism, Pfizer Worldwide Research & Development, La Jolla Laboratories, San Diego, CA, 92121, USA

**Keywords:** Androgen receptor, ChIP-Seq, Prostate cancer, AR antagonist, Molecular profiling

## Abstract

**Background:**

The androgen receptor plays a critical role throughout the progression of prostate cancer and is an important drug target for this disease. While chromatin immunoprecipitation coupled with massively parallel sequencing (ChIP-Seq) is becoming an essential tool for studying transcription and chromatin modification factors, it has rarely been employed in the context of drug discovery.

**Results:**

Here we report changes in the genome-wide AR binding landscape due to dose-dependent inhibition by drug-like small molecules using ChIP-Seq. Integration of sequence analysis, transcriptome profiling, cell viability assays and xenograft tumor growth inhibition studies enabled us to establish a direct cistrome-activity relationship for two novel potent AR antagonists. By selectively occupying the strongest binding sites, AR signaling remains active even when androgen levels are low, as is characteristic of first-line androgen ablation therapy. Coupled cistrome and transcriptome profiling upon small molecule antagonism led to the identification of a core set of AR direct effector genes that are most likely to mediate the activities of targeted agents: unbiased pathway mapping revealed that AR is a key modulator of steroid metabolism by forming a tightly controlled feedback loop with other nuclear receptor family members and this oncogenic effect can be relieved by antagonist treatment. Furthermore, we found that AR also has an extensive role in negative gene regulation, with estrogen (related) receptor likely mediating its function as a transcriptional repressor.

**Conclusions:**

Our study provides a global and dynamic view of AR’s regulatory program upon antagonism, which may serve as a molecular basis for deciphering and developing AR therapeutics.

## Background

Prostate cancer is the second most commonly diagnosed cancer and the second leading cause of cancer mortality in men in the United States. Despite decades of research, there are no effective treatment options available for the advanced stages of the disease. While androgen ablation therapy is a standard first-line treatment, the vast majority of prostate tumors eventually become hormone refractory and continue to proliferate even with very low levels of androgen. This stage, often referred to as castration-resistant prostate cancer (CRPC), is associated with an active androgen receptor (AR)-signaling pathway. Chen *et al.* reported that in human prostate cancer cell lines and xenografts derived from metastatic lesions, AR over-expression is necessary and sufficient to render the cells resistant to androgen withdrawal and antiandrogens [1]. The observation is further supported in the clinical setting where AR is frequently over-expressed in CRPC with AR amplification in up to 30% of those tumors [2-4].

AR, a member of the nuclear receptor (NR) superfamily, functions mainly as a ligand-dependent transcription factor. Upon binding of the androgenic hormone testosterone or its more active analog dihydrotestosterone (DHT) in the cytoplasm, AR translocates into the nucleus to bind DNA and regulate gene expression. AR has a wide range of regulatory roles in prostate growth and function, including but not limited to cellular proliferation, differentiation, apoptosis, metabolism and secretory activity [5]. While many of its direct activation targets have been characterized, the key downstream effectors, especially those playing a role in carcinogenesis or modulated during targeted therapy, remain to be determined; even less is known about the genes directly repressed by AR [6], though they may also be important contributors to AR function in disease and treatment settings.

Currently approved drugs aimed at androgen signaling axis include the AR antagonist bicalutamide and the CYP17 inhibitor abiraterone [7]. Given the critical role of AR in prostate cancer progression and particularly the late stages of the disease, additional therapeutic approaches are under development to target the receptor. Preclinical strategies involve double-stranded RNA interference, microinjection of anti-AR antibodies, and antisense oligonucleotides [2]. The most advanced agents in clinical testing are second-generation small molecule antagonists of AR function such as the diarylthiohydantoin MDV3100, which reduces the efficiency of AR nuclear translocation and impairs both DNA binding and recruitment of coactivators [8,9].

Recent advances in high throughput technologies such as ChIP-Chip and ChIP-Seq have enabled genome-wide identification of the AR cistrome in a number of preclinical models of prostate cancer [10-13]. While these studies provided novel insights into AR biology and gene regulatory networks, some important questions remain to be answered. In particular, the genomic landscape of AR binding has not been published in the presence of pharmacological agents, which are key to understanding the molecular activity of AR therapeutics. Furthermore, neither the core set of direct effector targets upon which AR’s binding and transcriptional activities are modulated by inhibitor drugs nor the oncogenic pathways they represent have been identified.

In this work, we employ chromatin immunoprecipitation coupled with massively parallel sequencing (ChIP-Seq) to provide the first publicly available genome-wide and dose-dependent inhibition map of AR binding by small molecules. By integrating sequence analysis, transcriptome profiling, cell viability assays and xenograft tumor growth inhibition studies, we explore the AR cistrome-activity relationship to render a global and dynamic view of its regulatory program upon small molecule antagonism. We also investigate endogenous and wild type AR binding at low androgen levels, a scenario that mimics prostate cancer patients following first-line androgen ablation therapy. Collectively, our study offers molecular insights into the pathological role of AR in CRPC progression and therapeutic-like contexts.

## Results

### A spectrum of genome-wide AR binding in VCaP cells

To create high-resolution, global maps of the interactions between DNA and androgen receptor, we profiled the VCaP cell line, which was derived from a vertebrate metastasis of a 59 year old male with CRPC. With high levels of endogenous wild type AR and TMPRSS2-ERG fusions as well as expression of many prostate epithelial markers, these cells serve as a useful model for CRPC tumor progression and metastasis [14,15]. VCaP cells were grown in the presence (+) or absence (−) of the synthetic AR agonist metribolone (R1881) to characterize AR binding in high and low androgen conditions respectively. Cross-linked chromatin from VCaP cells was immunoprecipitated with an antibody (H-280) highly specific for AR, which recognized a single major band at 110 kb on western blot and the same band was reduced by AR-siRNA treatment (Additional file 1: Figure S1). DNA pull-downs were then purified, amplified and sequenced with the Illumina Genome Analyzer 2, resulting in approximately 50 million single-end reads from each sample, which were then mapped to the most recent version of the human genome (hg19) with the ELAND algorithm.

Using Model-based Analysis of ChIP-Seq (MACS) [16], we identified 49998 and 15414 AR binding sites for R1881(+) and R1881(−) samples respectively. For subsequent analyses, we focused on the 16907 and 2307 high-confidence sites (Additional file 2: sFile 1), which had higher statistical significance than any of the “negative” peaks obtained by swapping the ChIP-Seq and control channels. The AR binding at all twelve tested regions was more than 3-fold above negative control by quantitative PCR analysis (Table 1), suggesting that the sites identified by ChIP-Seq represent *bona fide* AR binding. Additionally, the MACS binding (p-value) score was concordant (R = 0.87, P = 0.00025) with the enrichment values from qPCR.

**Table 1:** Real-time quantitative PCR (qPCR) validation of AR binding sites

**Genomic coordinates**			**Biding score (MACS)**	**Fold enrichment (MACS)**	**Fold enrichment over negative control (qPCR)**	**Primer sequence (forward)**	**Primer sequence (reverse)**
**chr**	**start**	**end**					
negative control						TGGACCTTTACCTGCTTTATCA	AGCAAGGACTAGGATGACAGAA
1	228856976	228857630		864.12	3.48	GAGGACACAACCCCATGACT	AGAGCGAAACTCCGTCTCAA
2	8808594	8809289		886.86	5.60	GATGGATGGATGGATGTCTT	CTGGTTTTCCAAGCTCACAA
2	237457017	237457622		633.63	8.63	GCAGGGAGGTCTTTGATCTG	TCCTGAATTGGTTTGCTCAT
3	187946846	187947612		665.42	8.93	CCCATTTGGCTTCTTACTTTGT	TTCCTTCCTGACTCCCACTG
4	175441647	175442310		986.29	23.03	CCAAAATATCATGTGCAATCAA	AAACACAATGCAAGAGGAACA
6	35699525	35700397			8.01	CGCATAGAAGCTAAGGGGAAAT	GATGTGAATGCAAGCCTGTC
6	43721200	43721909		1211.19	20.88	TGGCCTCTGTCTTTTGTGTT	CACAGCTTCCAACTAGCTTTACA
9	82188374	82188956		532.18	9.22	GTTGCGGGAGGAGAGTTTTA	GAAGCAGGGAGACGGAGAAA
10	3852210	3852832		500.44	12.13	CACCAGCTCCCAACTTTCAG	CAGCTTCCACTCCCTGTACC
19	51353679	51354605		3100	42.11	GTGTTGCTGTCTTTGCTCAG	CAGTGTTGGGAGGCAATTCT
20	35888865	35889525		511.25	3.10	GCAAGACCCCATCTCAAAGA	GGCTCGGCTACACTTCATTC
20	56260437	56261119		1605.03	35.60	CTGGCTGCTCCAGAGAACTA	CGGCCACGTACAGTCCTATT

12 AR binding sites identified from ChIP-Seq were tested for enrichment by real-time quantitative PCR. Reactions were carried out in triplicate. Negative control refers to a non-enriched region (a region in gene desert on chromosome 12).

**Figure 1:**
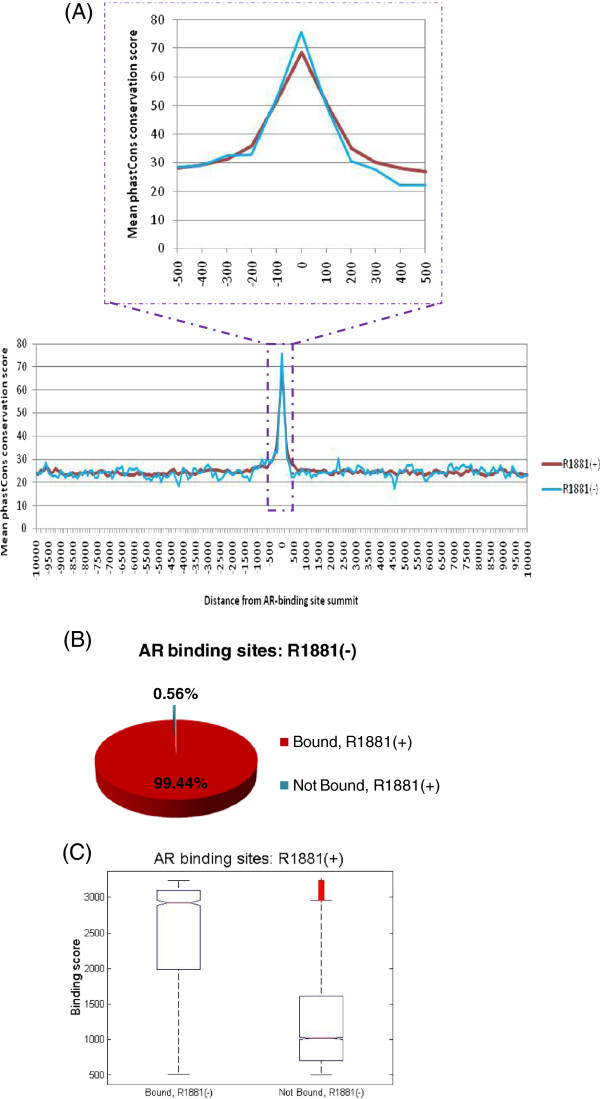
**Comparative analysis of AR binding in low and high androgen levels.** (**A**) Mean sequence conservation profiles based on phastCons score sampled every 100 bp from the summit of AR binding sites to 10 kb in both directions. The inset provides a zoom-in view of the profiles in the immediate vicinity of the summit. (**B**) Over 99% of the sites bound by AR in the absence of R1881 stimuli were also bound in its presence. (**C**) AR selectively occupied stronger binding sites in the absence of R1881 stimuli. AR binding sites defined from R1881(+) sample were divided into two groups based on overlap with R1881(−)-defined sites (Bound: n = 2330; Not Bound: n = 14577). Boxplots depict the distributions of their binding scores.

As functional elements tend to be evolutionarily conserved, we examined the multiple alignments of 45 vertebrate genomes to the human genome by sampling phastCons conservation score [17,18] every 100 bp. AR sites were most conserved at their binding summit and quickly dropped down to near genomic background level within 300 bp of either side of the summit (Figure 1A), underscoring the high resolution of ChIP-Seq technology as well as the accuracy of summit position calls by the MACS algorithm. Importantly, AR binding sites identified from R1881(−) sample were no less conserved than those from R1881(+) sample (Figure 1A), revealing that even with low levels of androgen, AR binding is far from random and likely occupies functional sites.

When AR binding sites were mapped to genomic annotations, they appeared only moderately associated with proximal promoters, with approximately 2 fold over-representation compared to genomic background (Additional file 3: Table S1). This is consistent with previous reports that AR often acts through distal enhancer elements [10,13,19]. Unbiased signature analysis showed that AR-bound genes were most significantly enriched with those transcriptionally regulated by the androgen receptor signaling pathway from mRNA profiling studies (Additional file 3: Table S2): depending on the exact expression signature, between 40% and 63% of the genes in the signature had high-confidence AR binding within 25 kb of their transcription start sites (TSS), whereas only 23% were expected.

We next performed a comparative analysis of AR binding in low and high androgen conditions. Strikingly, with more than 99% of AR binding sites identified in the absence of R1881 stimuli also bound in its presence (Figure 1B), the R1881(−) binding sites appeared to be a near-perfect subset of R1881(+) ones. Furthermore, the common binding sites were significantly biased towards those with higher binding score (P < 2.2e-16; Figure 1C and Figure 5C). Together, our findings reveal that even in low androgen level situations, such as those characteristic of androgen ablation treatment, AR is still functional by selectively occupying the strongest binding sites.

### AR binding and cell type

To investigate the role of cell type in AR binding, we compared sites identified in VCaP with those from other pre-clinical models of prostate cancer [10,11,13]. VCaP and LNCaP (including its androgen-independent derivative abl) cells share more than 60% of their AR binding sites regardless of the technology platform (ChIP-Chip or ChIP-Seq) used for profiling (Additional file 1: Figure S2). Interestingly, the overlap was even more extensive for those also occupied in the absence of R1881 stimuli (P_enrichment_ = 1.33e-192, 9.55e-219 and 2.64e-77 for LNCaP ChIP-chip, LNCaP-abl ChIP-chip and LNCaP Chip-seq respectively), implying that “baseline” AR binding tends to be preferentially conserved across cell types.

By contrast, AR binding in VCaP and PC3-AR cells were highly discordant and had only 41 sites in common, corresponding to 0.2% of total VCaP and 0.6% of total PC3-AR sites. Furthermore, we didn’t observe a significant enrichment of overlap for the R1881(−) subset (P = 0.19). As both datasets were collected using ChIP-Seq, this sharp divergence is more likely biological than technical: PC3 cells do not express androgen receptor endogenously and its AR binding was profiled following transfection of an AR construct [11]. Unlike the binding pattern in endogenous AR-expressing VCaP and LNCaP cells, the AR binding sites in PC3-AR cells were reported to be predominantly in the proximal vicinity of TSS and to lack androgen response elements (ARE) [11]. These differences underscore the important role that biological and experimental context plays in transcription factor binding and function.

### DNA cis-regulatory element associated with AR binding

A systematic search of known transcription factor binding motifs curated by the Genomatix MatBase database (http://www.genomatix.de) identified *cis*-regulatory elements for the GREF (Glucocorticoid responsive and related elements) family to be most enriched among AR-bound sequences (Additional file 3: Table S3), with 85% containing at least one copy of the motifs (Z-score = 131.43 and 41.33 for R1881(+) and R1881(−) samples respectively). The GREF family includes the androgen receptor and the closely related glucocorticoid, mineralocorticoid and progesterone receptors [20]. FKHD (forkhead domain factors) motifs were the second most over-represented family, consistent with previous reports [10,13] as well as its proposed role as a pioneer factor for AR [21]. Interestingly, neighboring GREF and FKHD elements (10–50 bp) had a clear distance preference at 15 bp (Additional file 1: Figure S3A), indicating a likely geometric constraint resulting from their interaction.

*Cis*-regulatory elements for many other AR interacting factors, such as GATA, HNF1 (Hepatic Nuclear Factor 1) and NF1F (Nuclear Factor 1), were also highly over-represented (Additional file 3: Table S3). Additionally, AR binding sites were enriched with sequence motifs recognized by the ABDB (Abdominal-B type homeodomain transcription factors) family (Additional file 3: Table S3), suggesting potential combinatorial control between androgen receptor and homeobox genes. HOXB13 has recently been reported to regulate the cellular response to androgens [22] as well as co-localize with AR to suppress androgen-stimulated PSA expression [23], while HOXC8 appears to negatively regulate AR signaling in prostate cancer cells by inhibiting SRC-3 recruitment to direct androgen target genes [24].

*Ab initio* motif discovery with the MEME algorithm [25] identified a perfectly palindromic 15 bp motif (Additional file 1: Figure S3B), supporting the observation that AR interacts with DNA as dimers [20,26]. It was highly specific to the AR-bound sequences [Z-score = 126.76 and 49.30 for R1881(+) and R1881(−) samples respectively; Figure 5E] and strongly resembled the androgen response element (ARE) described previously [12,13,27]. Furthermore, AR sites with this motif were stronger than those without (P = 2.27e-54; Additional file 1: Figure S3C), underscoring its role in determining AR binding.

### Small molecule inhibitors of AR function

**Figure 2:**
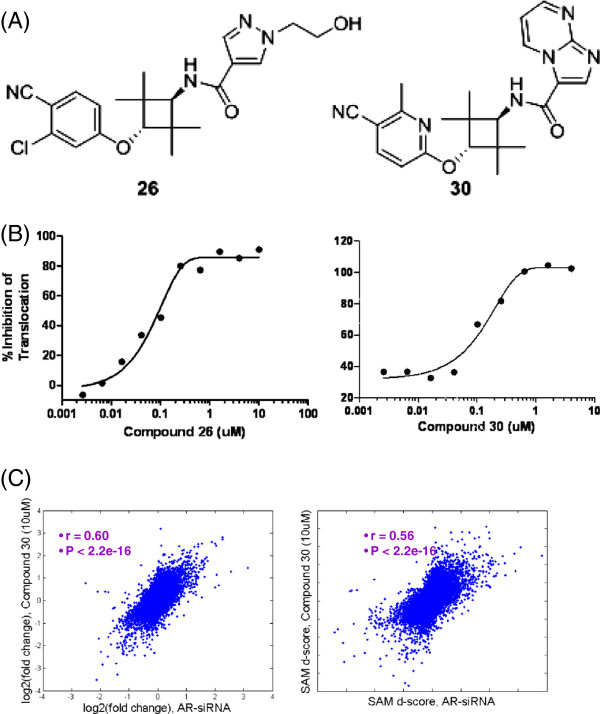
**Novel AR antagonists utilized in this study.** (**A**) Chemical structures (compound number listed below structure). (**B**) Nuclear Translocation of AR was impeded by these compounds. LNAR cells were treated with 0.1nM R1881 alone or in combination with the antagonist compounds at various doses to determine IC_50_ values. Nuclear translocation values were calculated as indicated under Methods. (**C**) Treatment of VCaP cells with small molecule AR antagonist induced similar genome-wide transcriptional effects as AR inhibition by siRNA. Left: fold change from the two types of treatments; Right: SAM d-score of differential expression from the two types of treatments.

After conducting an extensive structure-activity relationship (SAR) study using a CRPC cell-based high throughput screening, we identified two novel potent AR antagonists (Figure 2A) [28]. Importantly, these aryloxy tetramethylcyclobutane compounds had no agonist effect up to 10 μM concentration while effectively inhibiting AR translocation from the cytoplasm to the nucleus (Figure 2B and Table 2). Molecular modeling suggested that these compounds with relatively bulky substituents at the amide likely extend between Asn705 and Thr877 and force the critical Helix 12 of the AR ligand-binding domain (LBD) into a disrupted “open” conformation, thereby leading to AR full antagonism [28].

**Table 2:** Cell-based profile of AR antagonist compounds

	**AR agonism**		**AR antagonism**		**Nuclear**	**Cell**
	**(at 1uM)**				**Translocation**	**Proliferation**
	**FoldInduction**	**% Inhibition**	**IC50-nM**	**% Inhibition at 1uM**	**IC50-nM**	**IC50-nM**
Compound 30	0.83	-0.7	144	90	174	94
Compound 26	1.03	0.1	59	96	57	45

For agonism, values obtained from the AR antagonist compounds were compared to those of untreated cells, which were assigned an arbitrary number of 1.0 to indicate no agonism. For antagonism, cells were treated with 0.1nM R1881 alone (corresponding to max receptor activation = 100%) or in combination with the antagonist compounds at various doses to determine IC50 values. Nuclear translocation and cell proliferation values were calculated as indicated under materials and methods.

To confirm that AR is *de facto* the protein target of these compounds, we compared the mRNA profiles of VCaP cells treated with Compound 30 and those treated with AR-siRNA using the Affymetrix HG-U133Plus2.0 GeneChip array. Differential expression analysis was conducted with Significance Analysis of Microarray (SAM) algorithm [29]. The genome-wide Pearson correlation was 0.60 for fold changes and 0.56 for SAM d-scores (P < 2.2e-16 for both; Figure 2C), indicating a high degree of concordance between the two types of treatments. There was also striking overlap in significantly differentially expressed genes (P = 1.48e-11 for up-regulated and P = 4.52e-11 for down-regulated ones respectively). Thus, the small molecule antagonist induces similar global transcriptional effects as AR inhibition by siRNA.

**Figure 3:**
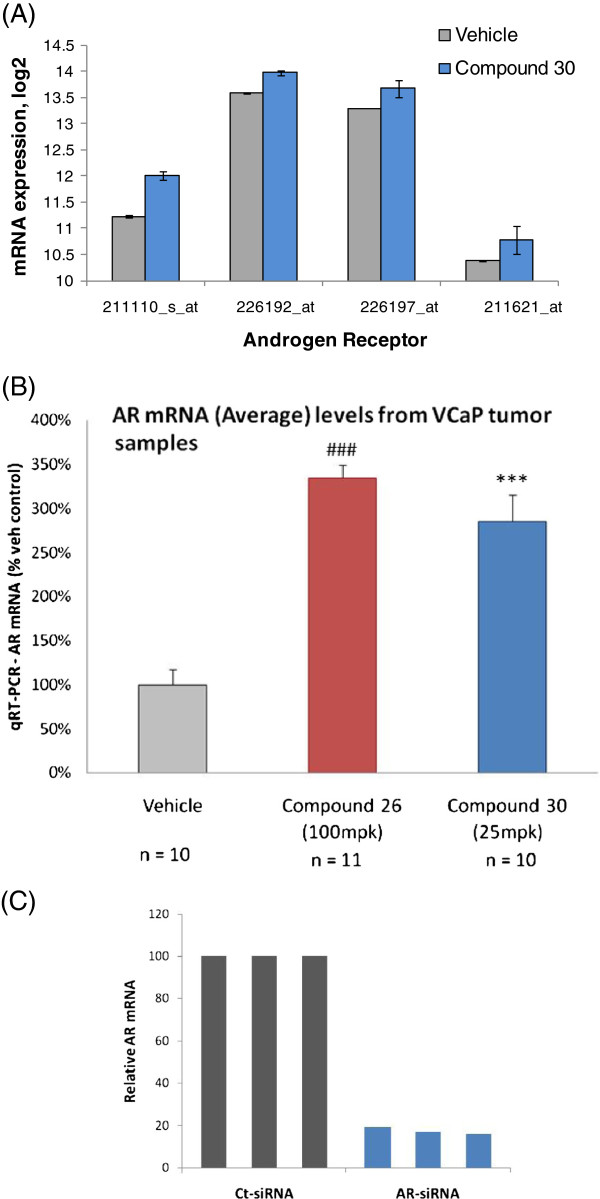
**Androgen receptor level increases upon small molecule antagonism.** (**A**) AR mRNA expression in VCaP cells (vehicle control and Compound 30, 10 μM) from four microarray probesets (The SAM q-value of differential expression are 0, 0.037, 0.043 and 0.056 respectively). The profiling experiment was performed using three independent biological replicates. (**B**) AR expression in tumors derived from VCaP cells implanted in CB17/lcr-Prkdc SCID mice and treated with Compounds 26 and 30 as measured by quantitative RT-PCR. n = number of animals per group; mpk = milligram per kilogram. Compound 26- and 30-treated groups were significantly different from Vehicle-group (###, *** P < 0.001). (**C**) AR expression in VCaP cells treated in triplicate for 48 hr with 25 nM of either control/non-targeted siRNA (Neg-siRNA, Dharmacon Cat# D-001810-10) or AR-siRNA pool (Dharmacon Cat# L-003400-00) as measured by quantitative RT-PCR. AR-siRNA treated samples were significantly different from control/non-targeted ones (P = 3.49e-5).

Interestingly, the mRNA level of the androgen receptor itself was notably higher in compound-treated cells compared to vehicle control across all four probesets for the gene on microarray (The SAM q-value of differential expression are 0, 0.037, 0.043 and 0.056 respectively; Figure 3A), suggesting that the cells respond to loss of AR by increasing its gene expression in a positive feedback loop. The microarray-based observation was further supported by RT-PCR measurements of AR expression in tumors derived from the VCaP cells implanted in mice (P < 0.001; Figure 3B), in sharp contrast to siRNA-treated cells where AR mRNA level was significantly reduced (P = 3.49e-5; Figure 3C).

### Effects on cell viability and tumor growth inhibition

**Figure 4:**
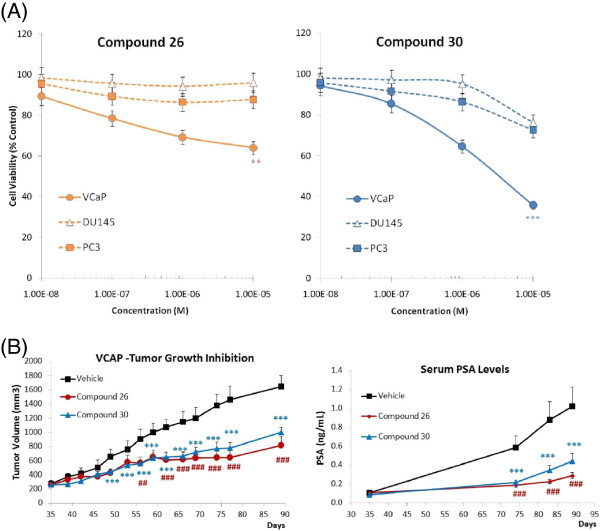
**Effect of AR antagonist treatment on prostate cancer cell viability and tumor growth inhibition.** (**A**) Number of live cells as a percentage of control treatment. Each data point represents the mean of at least three independent assays performed in duplicates. Bars represent standard deviation of the mean (SEM). * P < 0.05; ** P < 0.01; *** P < 0.001 (two-way ANOVA, GraphPad Prism). (**B**) Tumor Growth Inhibition (TGI) and PSA inhibition obtained from VCaP xenograft SCID mice treated for 3 months with Compounds 26 and 30. Tumor volume: The differences between both compound-treated groups and vehicle-treated control were statistically significant from Day 49 of treatment onward [Compound 30: *** P < 0.001 for all measurements; Compound 26: ## P < 0.01 at day 53 and ### P < 0.001 for the rest of the measurements). PSA levels: Compound 30-treated group was significantly different (*** P < 0.001) from vehicle-treated control on Days 74, 83 and 89; Compound 26-treated group was significantly different (### P < 0.001) from vehicle-treated control on Days 74, 83 and 90 (two-way ANOVA, GraphPad Prism).

To determine the impact and specificity of AR antagonist treatment on prostate cancer growth, we first assessed the effect of Compound 26 and 30 on cell viability using various pre-clinical models, including AR-positive VCaP cells and AR-negative DU145 and PC3 cells. Proliferation of cells treated in culture for up to seven days in the presence of these small molecule antagonists was significantly inhibited in VCaP compared to vehicle control, but was not significantly affected in those cells which do not express AR (Figure 4A), demonstrating that the antiproliferative effects elicited by the antagonist compounds were AR specific. Nevertheless, the behavior of the two compounds differed in VCaP cells: while Compound 30 dose-dependently inhibited cell proliferation, Compound 26’s impact plateaued at the highest concentration tested (10 μM).

### A genome-wide inhibition map of AR binding by small molecules

**Figure 5:**
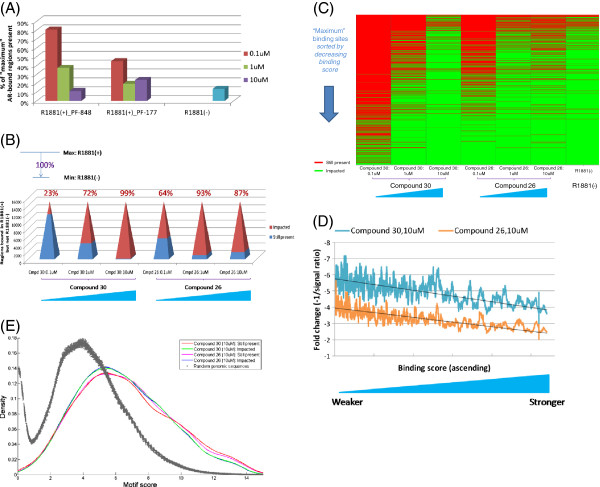
**AR binding upon small molecule antagonism.** (**A**) Number of high-confidence AR binding sites in various conditions. (**B**) Percent (%) impact of AR antagonists with increasing dosage. To quantify the molecular effects of AR antagonists, “maximum” and “minimum” AR binding were defined using non-antagonist-treated R1881(+) and R1881(−) cistromes and the % impact was based on their differentially occupied sites. (**C**) AR antagonists preferentially disrupted weaker binding sites. R1881(+)-defined binding sites were sorted by descending MACS binding score (in cases of a tie, they were further sorted by descending fold enrichment values), which approximates binding affinity. (**D**) AR antagonists had a greater effect on weaker binding sites. Fold changes were computed as −1/signal ratio and plotted as moving average with a window size of 100. Shown in black are linear trend lines. (**E**) Motif score distribution of the 15 bp perfect palindrome (Additional file: 1 Fig. S3B) for AR-bound sequences and 100 groups of randomly selected comparable sequences. The binding sites still occupied in the presence of the AR antagonists tend to have higher quality sequence motif (P < 0.01 for both compounds).

Finally, we profiled the AR cistrome in the presence of Compounds 26 and 30 at three different doses, 0.1 μM, 1 μM and 10 μM. Addition of the inhibitors reduced the number of AR binding sites compared to those of R1881(+) sample untreated by antagonist (Figure 5A). Consistent with their anti-proliferative behavior (Figure 4A), Compound 30 had a strong dose-dependent effect on AR binding while Compound 26’s impact saturated at 10 μM (Figure 5A), providing a direct molecular basis for deciphering the activity of these small molecule-based AR therapeutics.

To quantify the molecular effects of the two antagonists, we defined “maximum” and “minimum” AR binding using R1881(+) and R1881(−) cistromes in the absence of drug treatment respectively. The percent (%) impact measure was based on their differentially occupied sites. Strikingly, at 10 μM, Compound 30 achieved a 99% impact, reducing AR binding essentially from maximum to minimum level (Figure 5B) with a binding pattern similar to that of R1881(−) (Figure 5C). When sorted by their MACS binding score, a clear trend emerged that weaker sites were disrupted at lower dose (Figure 5C) and experienced greater changes (Figure 5D). Furthermore, the binding sites still occupied in the presence of the AR antagonists tend to have higher quality sequence motif of the 15 bp perfect palindrome that we identified (Figure 5E). To address the possibility that these patterns could arise because weaker binding sites are more prone to false positives, we included eleven sites from the lower half of the binding score spectrum for quantitative PCR analysis and they were all validated (Table 1).

The AR-antagonists were also evaluated for their *in vivo* efficacy in castrated VCaP tumor-bearing-CB17/lcr-Prkdc SCID mice treated with 25 mg/kg of Compound 30 and 100 mg/kg of Compound 26 daily by oral gavage. The compound doses were chosen to achieve average plasma exposure at least 10-fold higher than the target potency of the compounds (Table 3). As shown in Figure 4B, both AR antagonists effectively inhibited tumor growth and reduced PSA levels throughout the study (P < 0.001).

**Table 3:** ***In vivo *plasma concentrations of AR antagonist compounds on day 60, 4 hrs postdose, following daily oral administration**

	**Mouse Cp (nM)**		**Fold Free Cp/IC50**	
	**Total**	**Free**	**AR antagonism**	**VCaP proliferation**
**Compound 30 (25mpk)**	38946	2520	18	27
**Compound 26 (100mpk)**	11324	622	11	14

### A core set of direct downstream effectors modulated by AR antagonism

**Figure 6:**
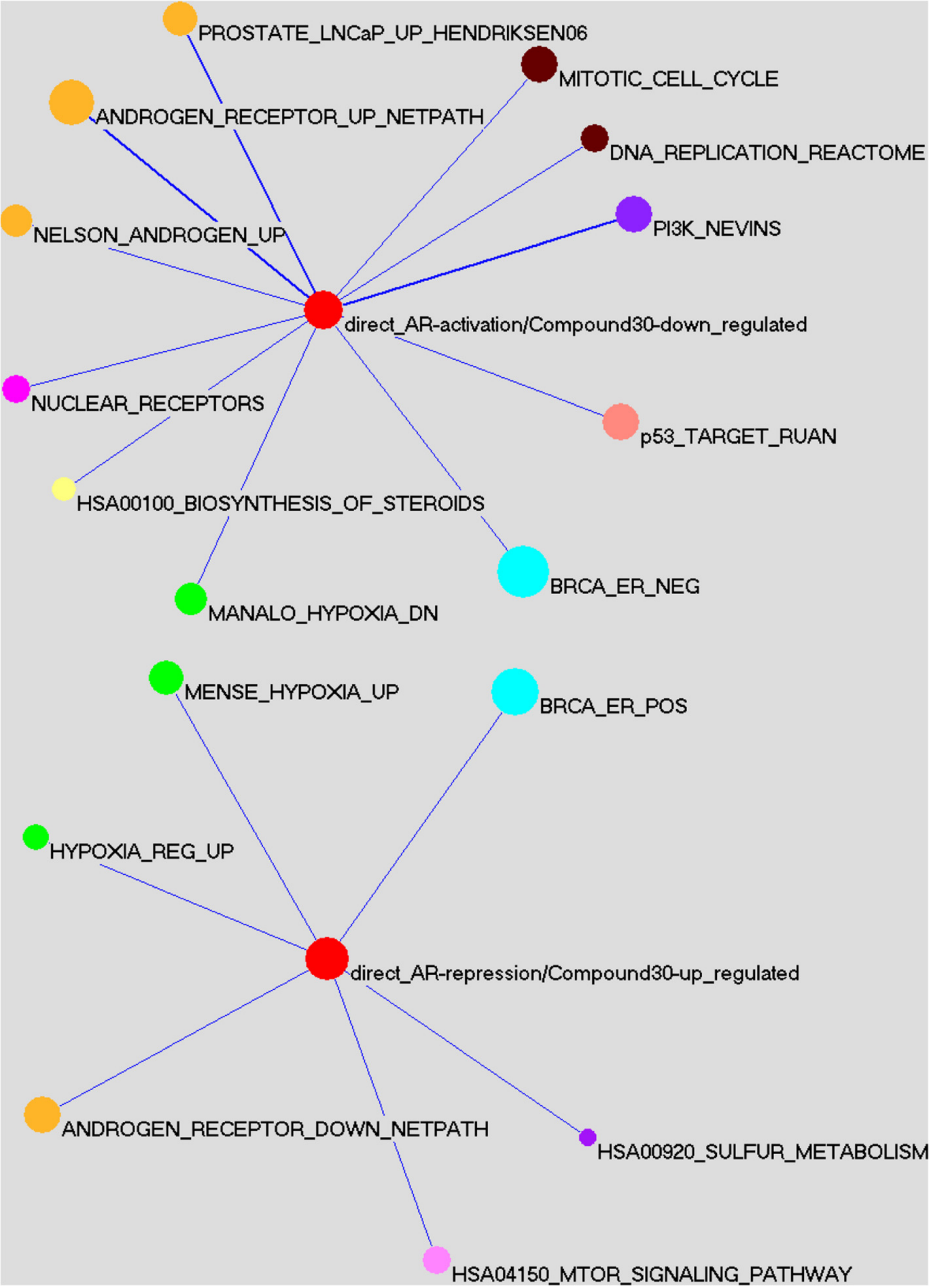
**Signature enrichment analysis of drug-modulated direct activation (top panel) and repression targets (bottom panel) of AR.** Shown are enriched gene signatures, with size of each node proportional to number of genes in the signature and width of each line proportional to statistical significance of the overlap between the signatures at the two ends. The signatures were colored by related biological concepts. Direct_AR-activation/Compound30-down_regulated targets refer to genes whose associated AR binding are impacted as well as mRNA level are significantly down-regulated upon Compound 30 treatment. Direct_AR-repression/Compound30-up_regulated targets refer to genes whose associated AR binding are impacted as well as mRNA level are significantly up-regulated upon Compound 30 treatment.

To obtain a multi-layer mechanistic understanding of the action of these AR modulators, we investigated the coupled expression change of those genes whose associated AR binding were impacted upon Compound 30 treatment. Notably, not only a significant number of them were down-regulated (P = 3.10e-10) but also many were up-regulated (P = 8.21e-4) in mRNA level upon small molecule antagonism, indicating that the compound influences direct AR targets from both modes of regulation. Our integrative analysis of cistrome and transcriptome data identified 195 direct activation targets and 306 direct repression targets of AR modulated by the drug-like compound respectively (Additional file 4: sFile 2). Although often overshadowed by its activation targets, the large number of direct repression targets suggests that androgen receptor also has a major role in negative gene regulation, which likely makes important contributions to its oncogenic mechanisms as well as the activities of targeted therapies. In fact, AR binding sites associated with direct repression targets were no less and even slightly more conserved than those associated with activation targets (P = 3.64e-2), underscoring their functional relevance. Systematic pathway mapping of drug-modulated direct AR target genes revealed that activation targets were over-represented in cell cycle, DNA replication, and steroids biosynthesis pathways, whereas repression targets were over-represented by those involved in hypoxia response, mTOR signaling and sulfur metabolism (Figure 6).

The direct activation targets of AR impacted upon antagonism include many members of its own nuclear receptor (NR) family (Figure 6; P = 9.60e-4) such as NROB1 (DAX1), NR2F1 (COUP-TF1) and THRB, revealing extensive crosstalk and potential hierarchical topology within the NR network. DAX1 has been reported to inhibit AR function [30] and there is a high-confidence physical interaction between the two proteins [31]. DAX1 is also known as a negative regulator of many genes in the steroid biosynthetic pathway [32,33]. Together, they suggest a feedback loop where an AR-DAX1 protein-protein interaction may serve to sense and prevent the over-production of DAX1 by AR while AR and DAX1 counter-balance each other’s effect on steroid synthesis (Additional file 1: Figure S4).

Emerging clinical data suggest that (metastatic) prostate tumors have increased expression of enzymes involved in steroid synthesis and lower levels of androgen inactivating enzymes compared to normal tissue [34]. As steroids are often inactivated by sulfation [35,36], our finding of direct regulatory links from AR to steroidogenesis and sulfur metabolism not only provides a mechanism underlying the observed gene expression changes in patient samples but also suggests an important new dimension to AR’s pathological function in CRPC. The down-regulation of steroid biosynthesis and up-regulation of sulfur metabolism by small molecule antagonists observed in this study suggests that these oncogenic activities of the androgen receptor can be relieved by targeted small molecule agents and may contribute to their therapeutic benefit in the clinic.

Interestingly, we observed a significant enrichment of the drug-modulated direct AR-activation targets among genes with higher expression in ER- breast tumors, while conversely, the direct AR-repression targets were significantly enriched among genes with higher expression in ER + breast tumors (Figure 6). Furthermore, estrogen response elements were disproportionately distributed towards binding sites near direct repression targets of AR compared to their activation counterparts (P = 0.0049; Additional file 3: Table S4). These point to a potential negative functional relationship between androgen and estrogen (related) receptors, where ER/ERR may mediate AR’s function in transcriptional repression.

## Discussion

Androgen receptor is a central player throughout development of prostate cancer, even after androgen deprivation therapy [2]. By comparing wild-type AR binding in the absence and presence of its ligand agonist metribolone, we found that AR bound to regulatory DNA elements even when androgen levels were low via selective occupancy of the strongest binding sites (Figure 1), offering molecular evidence for active AR signaling in CRPC tumors [2]. It complements other reported mechanisms for persistent AR signaling including receptor amplification or mutation [2-4,37,38], intratumoral conversion of weak adrenal androgens [39] and *de novo* steroid synthesis from cholesterol [40-42].

Previously published ChIP-Seq studies for androgen receptor [10-13] have focused on its binding in the absence of pharmacological intervention. Here, we characterize the dose-dependent effects of inhibition by drug-like small molecules on genome-wide AR binding: insights from this landscape can support the development of AR therapeutics because it provides a molecular basis for deciphering their pre-clinical and clinical activities. Both Compounds 26 and 30 (Figure 2A) are potent AR antagonists that also inhibit AR’s translocation from the cytoplasm to the nucleus (Figure 2B and Table 2). Interestingly, their molecular effects on the AR cistrome (Figures 5A and B) were consistent with corresponding phenotypic anti-proliferative behavior (Figure 4A), indicating a direct cistrome-activity relationship for these AR antagonists. Weaker sites or those with lower quality sequence motif of the 15 bp perfect palindrome appeared preferentially and more impacted (Figures 5C–E). Weaker transcription factor binding not only occurs abundantly *in vivo* but may also be functionally important features of the genomic regulatory program as revealed by evolutionary and gene expression analyses [43]. Our observations here further suggest that they may also be relevant in a therapeutic context and underscore the need to look beyond the strongest binding sites.

Given that our drug-like inhibitors act exclusively as AR antagonists, we not only identified a core set of direct downstream effector genes for androgen receptor by integrating cistrome and transcriptome profiling data upon compound treatment, but also characterized their associated mode of regulation (Additional file 4: sFile 2). Importantly, these are candidate mediators in a therapeutic setting since both AR’s binding and transcriptional activities at these loci were modulated by small-molecule antagonists. Unbiased pathway mapping further revealed AR as a key regulator of steroidogenesis (Figure 6). Emerging data indicates that prostate tumor cells are capable of synthesizing their own androgens to sustain growth [44]: for instance, the expression of enzymes involved in *de novo* steroid synthesis has been reported to be up-regulated in both (metastatic) prostate tumors [34] and CRPC patients after CYP17A1 inhibitor treatment [40]. We found AR directly regulates several key players (Additional file 3: Table S5), a novel oncogenic mechanism that would be relieved by antagonist treatment. Thus our result supports the recently proposed combination therapy strategy of treating with CYP17A1 and AR inhibitors in a concurrent or sequential manner [44].

AR also appears to directly and positively modulate the expression of its own nuclear receptor family (Figure 6): the most notable is NROB1 (DAX1), an orphan nuclear receptor and a global regulator of hypothalamic-pituitary-adrenal/gonadal axis (HPAG) ontogenesis and steroidogenesis [32]. DAX1 inhibits the activity of steoidogenic factor (SF-1) by directly binding to its own promoter, preventing SF-1 mediated transcription and hence interfering with hormone synthesis [30]. DAX-1 also prevents ligand-activated AR from being imported into the nucleus via a nucleocytoplasmic shuttling mechanism. Together with our observations above, AR and DAX1 appear to form a tightly controlled feedback loop in controlling steroid biosynthesis (Additional file 1: Figure S4). To add to the complexity of the AR-DAX1-steroidogenesis network, it has also been reported that the DAX1 promoter has a redundant region that creates a binding site for SF-1 and NR2F2 (COUP-TF1) [45], the latter of which is another NR member identified as a direct AR activation target in our current study (Figure 6).

The drug-impacted direct repression targets of AR were significantly enriched with those related to sulfur metabolism (Figure 6) such as SULT2B1 and PAPSS1 (Additional file 3: Table S5). SULT2B1 is involved in sulfation of the steroids dehydroepiandrosterone (DHEA) and delta(5)-androstenediol [Delta(5)-Adiol] to prevent their conversion to more potent androgens/estrogens, and its inhibition leads to increased cell proliferation [35]. PAPSS1 is an enzyme essential to synthesize activated sulfate donor (3’-phosphoadenosine 5’-phosphosulfate, PAPS), a cofactor that inactivates steroid hormones through sulfation [46,47]. Also directly repressed by AR is ACOX2 (Additional file 3: Table S5), a branched-chain acyl-CoA oxidase enzyme that takes part in the degradation of long branched fatty acid and bile acid intermediates in peroxisomes and is down-regulated in castration-resistant prostate cancer [48]. These observations are particularly interesting given the clinical data that metastatic prostate cancer express lower levels of androgen inactivating enzymes [34] and complement the result described above that AR positively regulates the expression of many genes involved in biosynthesis of steroids.

We found an inverse functional relationship between androgen and estrogen (related) receptors in VCaP cells, with ER/ERR likely contributing to AR’s role as a transcriptional repressor (Figure 6, Additional file 3: Table S4). Whereas AR has recently been reported to inhibit ER [49], our study reveals a novel reciprocal effect, providing further support for crosstalk and counter-balance between the two families of sex steroid hormone receptors. It remains to be determined whether ER/ERR contributes to AR repression by direct competition for DNA binding or through interaction with its cofactors. In addition to the well-established association of ERs with breast cancer, emerging data suggest that they also play important roles in prostate cancer. For instance, ERβ, localized in prostate epithelial cells together with AR and DAX1, is pro-apoptotic, anti-proliferative and anti-inflammatory and impedes prostate cancer EMT [50,51]. ERβ agonists were shown to activate apoptosis through tumor necrosis factor-α (TNF-α) signaling and target cells that are resistant to systemic androgen deprivation [52]. Additionally, the closely related orphan nuclear receptor ERRβ has been reported to be down-regulated in prostate cancer cells and carcinoma lesions and it performs as a tumor suppressor [53]. While stable ERRβ expression suppressed *in vivo* prostate tumor growth, treatment with an ERR agonist potentiated ERRβ-induced growth inhibition of prostate cancer cells. Lately the many similarities between breast and prostate cancer have become widely appreciated [49], leading to important therapeutic implications, such as a phase II clinical trial that is currently underway to investigate the potential benefit of targeting AR in triple negative breast cancer [54].

Our observation that the expression of the drug target itself (androgen receptor) was significantly up-regulated upon small molecule antagonism (Figures 3A and B) points to an interesting feedback loop of how cells react to AR inhibition. This pattern appears to be a recurrent theme in molecular drug responses: for example, very recently it was reported that CYP17A1 level was markedly increased in tumor biopsies from CRPC patients after CYP17A1 inhibitor therapy [40]. Further characterization of this control structure, especially in terms of network properties, may elucidate a general mechanism underlying antagonist drug response and associated clinical outcome.

## Conclusions

Our study charts the dose-dependent effects of small-molecule antagonists on the genomic landscape of AR binding and elucidates their relationship with phenotypic and transcriptional activities. These novel insights into modulation of the AR regulatory program upon therapeutic antagonism provide a molecular platform for deciphering and developing next generation of pharmacological agents targeting the androgen receptor.

## Methods

### Accession numbers

The NGS and microarray data have been deposited in the NCBI Sequence Read Archive (SRA) and Gene Expression Omnibus (GEO) [SRA: SRP008849, GEO: GSE32892].

### Cell culture

VCaP human prostate cell lines were obtained from ATCC and grown in DMEM (GIBCO Cat# 11995) 10% FBS (SIGMA Cat# 12103 C-500 ml). Medium was supplemented with standard antibiotics (Penicillin-Streptomycin, GIBCO #15070-063).

### Transactivation assay

AR trans-activation assay was performed as previously described [28]. Briefly, LNCaP cells were engineered to over-express wild type human AR and to express an ARE2-PB-Luc reporter (LNAR cells). Cells were starved for 3 days prior to performing trans-activation assays, in phenol red free (PRF)-RPMI Medium supplemented with 5% of charcoal stripped FBS. On the day of the assay, cells were seeded at a density of 5,000 cells/well in 96 well plate in starvation medium and 4 hr later treated with the compounds in the absence (agonistic mode) or presence (antagonistic mode) of 100pM R1881 for 24 hr. Luciferase readings were acquired by means of a Perkin Elmer EnVision Excite Multilabel Reader (Ultra Sensitive Luminescence method).

### AR nuclear translocation

LNAR cells were starved for two days in phenol red free RPMI medium containing 5% charcoal-dextran stripped FBS (Omega Scientific) prior to the assay. For Nuclear Translocation (NT) assay, 3,000 cells per well (in 384 well plates) were treated with compounds and 100pM R1881. Following overnight incubation the cells were fixed with 10% Formalin and permeabilized with PBS containing 0.5% Triton X-100 and blocked with 1% BSA. Cells were then stained with anti-AR monoclonal antibody (Abcam) followed with alexa 488 conjugated anti-Mouse IgG secondary reagent (Invitrogen). Finally the cells were counter stained with Hoechst 33342 (Invitrogen) and Cell mask Deep Red (Invitrogen). Plates were sealed and imaged using an Evotec Opera high content imager. Images were analyzed using an Acapella (Evotec) algorithm customized by Pfizer to quantify the fluorescence associated with anti-AR in the cytoplasm and nuclear regions. The ratio of Nuclear to Cytoplasm fluorescence was calculated and used as to tract inhibition of AR translocation.

### Cell viability

Cells were seeded at a density of 15,000 cells/well (VCaP) or 1,000 cells/well (PC3 and DU145) in 96 well-plates and treated after attachment to the plate with test compounds. Medium and compounds were refreshed every 2–3 days. Number of live cells was analyzed at day 7 using the Resazurin assay (SIGMA Cat# R7017).

### Other treatments

Cells were starved for 3 days in phenol red free DMEM containing 5% charcoal stripped FBS, and then seeded in 6-well plates at a density of 1 million cells per well in starvation medium. In the case of compound treatment (for ChIP-Seq study), cells were allowed to attach overnight and then were treated with various doses of the AR antagonists or vehicle alone (0.1% DMSO) in the absence or presence of 1nM R1881. Cells were incubated for 30 min at 37°C, 5% CO_2_ and then processed.

For siRNA treatment, cells were seeded in DMEM containing 10% FBS and transfected the day after seeding with 25nM AR-siRNA pool (Thermo Scientific, L-003400-00-0020) using Lipofectamine 2000 reagents (Invitrogen, 11668–019) and following manufacturer’s instructions. Cells were incubated at 37°C 5% CO_2_ for 48 h and then processed.

In both procedures, after the indicated treatment time, cells were rinsed once with ice-cold PBS and then lysed with Qiagen RNeasy Plus Kit (Cat# 74134, Qiagen, Valencia, CA). RNA quality was assessed using the Bioanalyzer (Agilent, Sunnyvale, CA) and spectrophotometer.

### Chromatin immunoprecipitation (ChIP)

ChIP was carried out by Active Motif (formerly Genpathway) as follows. Cells were fixed with 1% formaldehyde at room temperature for 15 minutes. Fixation was stopped by the addition of glycine to a final concentration of 0.125 M glycine. Chromatin was isolated from the sample by adding 10 ml lysis buffer containing PIPES, Igepal, PMSF and Protease Inhibitor Cocktail, followed by disruption with a Dounce homogenizer. Samples were pelleted by centrifugation and resuspended in buffer containing sodium deoxycholate, SDS, and Triton X-100. Lysates were sonicated using a Misonix Sonicator 3000 equipped with a microtip in order to shear the DNA to an average length of 300–500 bp. Lysates were cleared by centrifugation and the chromatin suspensions were transferred to new tubes and stored at −80°C. To prepare Input DNA (genomic DNA), two aliquots of 10 μl each (approximately 1/50 of each chromatin preparation) were removed and treated with RNase for 1 hr at 37°C, proteinase K for 3 hr at 37°C, and 65°C heat for at least 6 hr to overnight for de-cross-linking. DNAs were purified by phenol-chloroform extraction and ethanol precipitated. Pellets were resuspended in 1/5 TE buffer. Resulting DNAs were quantified on a Nanodrop spectrophotometer. Extrapolation to the original chromatin volume allowed determination of the yield for each chromatin preparation (as measured by the DNA content).

Prior to use in ChIP, protein A agarose beads (Invitrogen) were pre-blocked using blocking proteins and nucleic acids for 3 hr. For each ChIP reaction, an aliquot of chromatin (30 μg) was pre-cleared with 30 μl pre-blocked protein A agarose beads for 2 hr. ChIP reactions were set up using pre-cleared chromatin and antibody AR (Santa Cruz Biotechnology, Cat# sc-13062, Lot# D0610) in a buffer containing sodium deoxycholate and incubated overnight at 4°C. Pre-blocked protein A agarose beads were added and incubation at 4°C was continued for another 3 hr. Agarose beads containing the immune complexes were washed two times each with a series of buffers consisting of the deoxycholate sonication buffer, high salt buffer, LiCl buffer, and TE buffer. An SDS-containing buffer was added to elute the immune complexes from the beads, and the eluates were subjected to RNase treatment at 37°C for 20 min and proteinase K treatment at 37°C for 3 hr. Cross-links were reversed by overnight incubation at 65°C, and ChIP DNAs were purified by phenol-chloroform extraction and ethanol precipitation. Quality of ChIP enrichment was assayed by qPCR using primers against known positive control site(s). Input DNA was queried at the same sites in parallel.

### Sequencing

ChIP DNA was amplified by following the Illumina ChIP-Seq DNA Sample Prep Kit protocol. In brief, DNA ends were polished and 5’-phosphorylated using T4 DNA polymerase, Klenow polymerase and T4 polynucleotide kinase. After addition of 3’-A to the ends using Klenow fragment (3’-5’ exo minus), Illumina genomic adapters were ligated and the sample was size-fractionated (200–250 bp) on a 2% agarose gel. After a final PCR amplification step (18 cycles, Phusion polymerase), the resulting DNA libraries were quantified and tested by QPCR at the same specific genomic regions as the original ChIP DNA to assess quality of the amplification reactions. DNA libraries were sequenced on a Genome Analyzer II.

### Identification of AR binding sites

Alignment of the 36-bp single-read sequences (“tags”) from ChIP-Seq to the human genome (hg19) was conducted by Active Motif with ELAND (Illumina CASAVA 1.5 pipeline) software. Tag density was calculated by dividing the genome into 32-nt bins and counting the number of 3’-end extended tags in each bin (Active Motif). Only sequence reads that pass quality filtering, with an alignment score of at least 10 and perfect genomic match were included for peak detection. AR-enriched genomic regions (binding sites) were identified by comparing the ChIPed samples with input sample using MACS algorithm [16] (1.4.0rc 2) and option of “-p 1e-10”. For subsequent analyses, we used the most high-confidence regions (FDR < 0.01) based on joint p-value score and fold enrichment cutoffs of 500 and 20. The values were chosen in consideration of “negative” peaks generated from swapping the ChIP-Seq and control channels (These “negative” peaks have no biological meaning and thus serve as a control for estimating/filtering out technical noises). To enable quantitative comparison (e.g. fold change) of the same binding site across samples, a “signal” measurement was computed for it in each sample by combining tag density values for bins that fall within the binding site with one-step Tukey's biweight algorithm.

### Quantitative PCR (qPCR) validation

Twelve AR binding sites identified from ChIP-Seq were tested for enrichment by real-time quantitative PCR. Reactions were carried out in triplicate. Fold enrichment was determined relative to a non-enriched region (a region in gene desert on chromosome 12). Their primer sequences were included in Table 1.

### Mapping to genomic annotations

AR binding sites were mapped to transcriptional start sites (TSS) of genes based on refFlat (hg19) table from UCSC Genome Browser. The classification of AR binding sites relative to genomic annotations (promoter/exomic/intronic/intergenic) and calculation of associated enrichment statistics were performed with RegionMiner tool and ElDorado database (Genomatix).

### Sequence conservation analysis

Sequence conservation was assessed using phastCons conserved elements [17,18] derived from multiple alignments of 45 vertebrate genomes to the human genome. Conservation score was sampled every 100 bp from the summit of each AR binding site (as reported by MACS) to 10 kb in both directions. It was defined to be the phastCons score of the overlapping conserved element, or zero for those outside of conserved elements. To explore the relationship between sequence conservation and mode of AR regulation, binding sites were classified in a binary fashion as conserved or non-conserved based on summit position. Statistical significance of the association was determined using two-tail Fisher’s exact test.

### Motif analysis

AR bound-sequences were searched for predefined motif matrices of transcription factors from MatBase library v8.3 vertebrate collection using RegionMiner (Genomatix). Over-representation statistics were reported as Z-score (the distance from the population mean in units of the population standard deviation) computed against genomic background (NCBI37/hg19). V$GREF-V$FKHD pair (module) is defined as two elements from 10 to 50 bp (middle to middle) of each other. Their occurrences were examined for distance distribution within the range.

MEME algorithm [25] was used to discover enriched sequence motifs *ab initio* from repeat-masked AR-bound sequences. In cases where a binding site is longer than 500 bp, only 500 bp centered on its summit were used. In consideration of computational time, we preformed the search with 2500 top sequences in terms of binding score. MEME was run using “-dna -mod zoops -revcomp -evt 0.01” command line options. Specificity was assessed as Z-score from 100 randomly sampled groups of the same number of sequences of the same length from the same chromosomes as AR binding sites. To investigate the enrichment and score distribution of the above MEME-derived ARE consensus motif, we scanned the AR-bound sequences as well as randomly sampled genomic sequences with its position weight matrix using PATSER [55] (v. 3e) and command line options “-c -li -s u2” or “-c -ls 0 -s -u2”. We determined presence/absence of motifs as predefined vertebrate matrices from MatBase in a similar manner (PATSER and “-c -li -s u2” option), whereas their statistical association with mode of AR regulation (direct activation/repression) was computed using two-tail Fisher’s exact test.

### Quantitative Reverse Transcriptase- PCR (qRT-PCR) – *in vivo* samples

Approximately 20 mg of tumor samples in RNALater were homogenized by means of Qiagen TissueLyser 2, for 2 min @ 20 Hz. Homogenates were then processed using Qiagen RNeasy Plus kit (Cat#74134). Samples were resuspended in 60μL water and 2 μg RNA from each sample were subsequently subjected to qRT-PCR using TaqMan RT-PCR ABI7900HT, in a two-step procedure, as following: 1) For reverse transcriptase step we utilized ABI High Capacity cDNA Reverse Transcription Kit (4368814,from Applied Biosystems). Cycle run was 10 min 25°C, 2 h 37°C, 5 min 85°C and cool down to 4°C. For the qPCR reaction we utilized ABI 2X universal Master mix (4324018, Applied Biosystems) using ddCt (RQ) method for quantification. Cycle was 10 min 95°C, 15 s 95°C, 60s 60°C, 40 cycles. Primers used were ID# Hs00907244-ml for for AR and # 4352934E for GADPH (Applied Biosystems).

### Tumor growth inhibition (TGI) – VCaP CRPC *in vivo* model

VCaP (3.5million) cells (in 50% matrigel product# 354234, lot A7141) were implanted subcutaneously in CB17/lcr-Prkdc SCID mice, and when tumors reached about 200 mm^3^ in size the animals were castrated. Since PSA levels remain low in this *in vivo* model until the tumors are significantly large (400 mm^3^), the re-growth of tumors post-castration was interpreted as a sign that the animals entered into the castration refractory phase. Animals were then randomized based on tumor volume and treatment commenced. In general and unless otherwise indicated, compounds were given by oral gavage once daily. Vehicle formulation consisted of 0.9% benzyl alcohol, 1% Tween-80 and 98.1% methylcellulose (0.5%). Tumor volume was calculated by the formula: length x width x depth x 0.5236. To measure PSA, 15 μl serum was diluted 1:3 v/v in water and then 25 μl of the dilution samples were transferred to the ELISA plate for the assay (PSA ELISA Kit from American Qualex Antobodies, Cat# KD4310). At the end of the study, animals were sacrificed and the tumors were extracted and treated with RNALater (Qiagen, Cat# 76154). Statistic analysis was performed using two-way ANOVA (GraphPad Prism, Version 5.01 - http://www.graphpad.com).

### Expression profiling and data analysis

RNA from VCaP cells treated with AR-siRNA, Compound 30 (10 μM) or corresponding controls underwent 1st and 2nd strand cDNA synthesis, *in vitro* reverse transcription, and target preparation following the GeneChip Expression Analysis Technical Manual (Affymetrix). Overnight hybridization of the fragmented cRNA on the GeneChip® Human Genome U133A 2.0 array and subsequent washing, staining and scanning steps were performed as suggested by the manufacturer (Affymetrix). Image analysis was done with the Expression Console (Affymetrix).

Expression profiling data were RMA normalized with “affy” package of Bioconductor, followed by exclusion of spike-in controls (AFFX) and mixed cross-hybridization (_x) probe sets. Significance Analysis of Microarray (SAM) algorithm [29] implemented in “samr” package was used for differential expression analysis between compound/siRNA-treated and control samples. The fold change (FC) and d-score outputs from all probe sets were used for computation of genome-wide correlations. Significantly differentially expressed genes refer to those with FDR < 0.05 and |FC| > 1.5. Genes with probe sets going opposite directions were not included in subsequent analyses.

### Gene signature enrichment analysis

Gene signature enrichment analysis was performed by comparing direct AR- activation/repression targets from small molecule antagonism with signatures collected from a variety of public databases and studies (e.g. MSigDB, GeneSigDB, NetPath, Gene Ontology, KEGG). Statistical significance of signature enrichment was determined using cumulative hypergeometric probability distribution as previously described [56] and correction for multiple hypothesis testing was conducted with the Q-value package [57]. Some significantly enriched signatures and their connections were plotted with network visualization tool Pajek [58]. We only reported enriched signatures with corresponding FDR < 0.05.

## Abbreviations

AR: Androgen receptor; CRPC: Astration-resistant prostate cancer; ChIP-Seq: Chromatin immunoprecipitation coupled with massively parallel sequencing; TSS: Transcriptional start site; ARE: Androgen response element; SAR: Structure-activity relationship; TGI: Tumor growth inhibition.

## Competing interests

All authors are or were employees of *Pfizer Inc*. at time of performing the studies described herein.

## Authors’ contributions

AF and GL conceived the experimental plan; ZZ and PR designed the analysis plan; MS and AF conducted cell treatment and ChIP-Seq profiling; ZZ conducted ChIP-Seq and integrative data analysis; WH conducted mRNA profiling; ZZ, HE, WH conducted microarray expression data analysis; MS conducted qRT-PCR experiment of AR expression; MS and AF conducted cell viability study; DB and AF conducted TGI and PSA inhibition study; JE conducted AR trans-activation study; NH conducted AR antagonist pharmacokinetic study; TN conducted AR nuclear translocation assay and KI67 IHC study; ZZ, AF, PR wrote and revised the manuscript. All authors read and approved the final manuscript.

## Supplementary Material

Additional file 1 **Figure S1.** AR protein expression. VCaP cells were treated in the presence of 25nM control/non-targeted siRNA or AR-siRNA pool. The protein levels of AR were analyzed by western blot using anti-AR (Santa Cruz Cat# sc-815). Anti-Tubulin (Santa Cruz, Cat# sc-12462-R) was included for loading control. **Figure S2.** AR binding and cell type. Overlap of the AR binding sites between VCaP cells and other cell types from previous studies: ^1^(Lin *et al.*, 2009); ^2^(Wang *et al.*, 2009); ^3^(Massie *et al.*, 2011). Binding sites based on earlier version of human genome were remapped to hg19 using UCSC liftOver tool. Overlap was defined by sharing of at least 1 bp. **Figure S3.** AR binding and sequence features. (A) Distance distribution between neighboring (10-50 bp) GREF and FKHD elements. (B) The motif identified *de novo* from AR-bound sequences appears to be a 15 bp perfect palindrome. (C) Motif and binding strength. AR binding sites were divided into two groups based on whether they had a significant occurrence of the palindromic motif in (B). Boxplots depict the distributions of their binding scores. **Figure S4.** AR and DAX1 form a tightly controlled feedback loop on steroid biosynthesis: AR and DAX1 counter-balance each other’s effect on steroidogenesis. AR also directly and positively regulates the expression of DAX1, whereas their physical interaction may serve to sense and prevent the over-production of DAX1 by AR. Dashed links refer to previously reported regulatory relationships, while solid links describe regulatory relationship identified in this study. Positive or stimulatory effects are represented by (+), and negative or inhibitory effects are represented by (−). Click here for file

Additional file 2 **sFile1.** AR binding sites list. Click here for file

Additional file 3 **Table S1.** The distribution of AR binding sites relative to genomic annotations. **Table S2.** Gene signatures most enriched among AR-bound genes. **Table S3.** MatBase families most over-represented among AR-bound sequences, sorted by descending Z-score. **Table S4.** Transcription factor binding motifs associated with mode of AR regulation. **Table S5.** Selective drug-modulated direct downstream effectors of AR involved in steroid metabolism. Click here for file

Additional file 4 **sFile2.** Drug-modulated direct activation and repression targets of AR from small molecule antagonism. Click here for file
