# Supplementary material for: Dose-dependent effects of small-molecule antagonists on the genomic landscape of androgen receptor binding
Source: BMC Genomics. 2012 Jul 31;13:355. doi: 10.1186/1471-2164-13-355 (PMC3507642; doi:10.1186/1471-2164-13-355)
Supplement: Additional file 1 — Figure S1. AR protein expression. VCaP cells were treated in the presence of 25nM control/non-targeted siRNA or AR-siRNA pool. The protein levels of AR were analyzed by western blot using anti-AR (Santa Cruz Cat# sc-815). Anti-Tubulin (Santa Cruz, Cat# sc-12462-R) was included for loading control. Figure S2. AR binding and cell type. Overlap of the AR binding sites between VCaP cells and other cell types from previous studies: 1(Lin et al., 2009); 2(Wang et al., 2009); 3(Massie et al., 2011). Binding sites based on earlier version of human genome were remapped to hg19 using UCSC liftOver tool. Overlap was defined by sharing of at least 1 bp. Figure S3. AR binding and sequence features. (A) Distance distribution between neighboring (10-50 bp) GREF and FKHD elements. (B) The motif identified de novo from AR-bound sequences appears to be a 15 bp perfect palindrome. (C) Motif and binding strength. AR binding sites were divided into two groups based on whether they had a significant occurrence of the palindromic motif in (B). Boxplots depict the distributions of their binding scores. Figure S4. AR and DAX1 form a tightly controlled feedback loop on steroid biosynthesis: AR and DAX1 counter-balance each other’s effect on steroidogenesis. AR also directly and positively regulates the expression of DAX1, whereas their physical interaction may serve to sense and prevent the over-production of DAX1 by AR. Dashed links refer to previously reported regulatory relationships, while solid links describe regulatory relationship identified in this study. Positive or stimulatory effects are represented by (+), and negative or inhibitory effects are represented by (−). [file 1471-2164-13-355-S1.pdf]

Figure S1

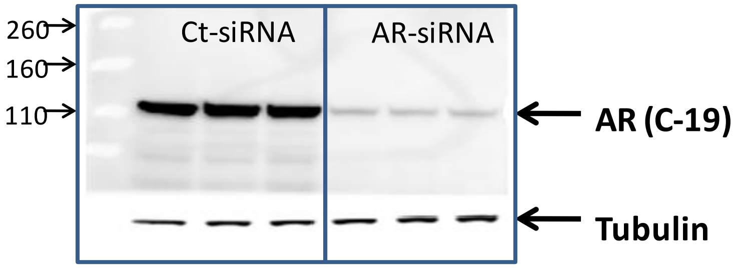

AR protein expression. VCaP cells were treated in the presence of 25nM control/non-targeted siRNA or AR-siRNA pool. The protein levels of AR were analyzed by western blot using anti-AR (Santa Cruz Cat# sc-815). Anti-Tubulin (Santa Cruz, Cat# sc-12462-R) was included for loading control.

Figure S2

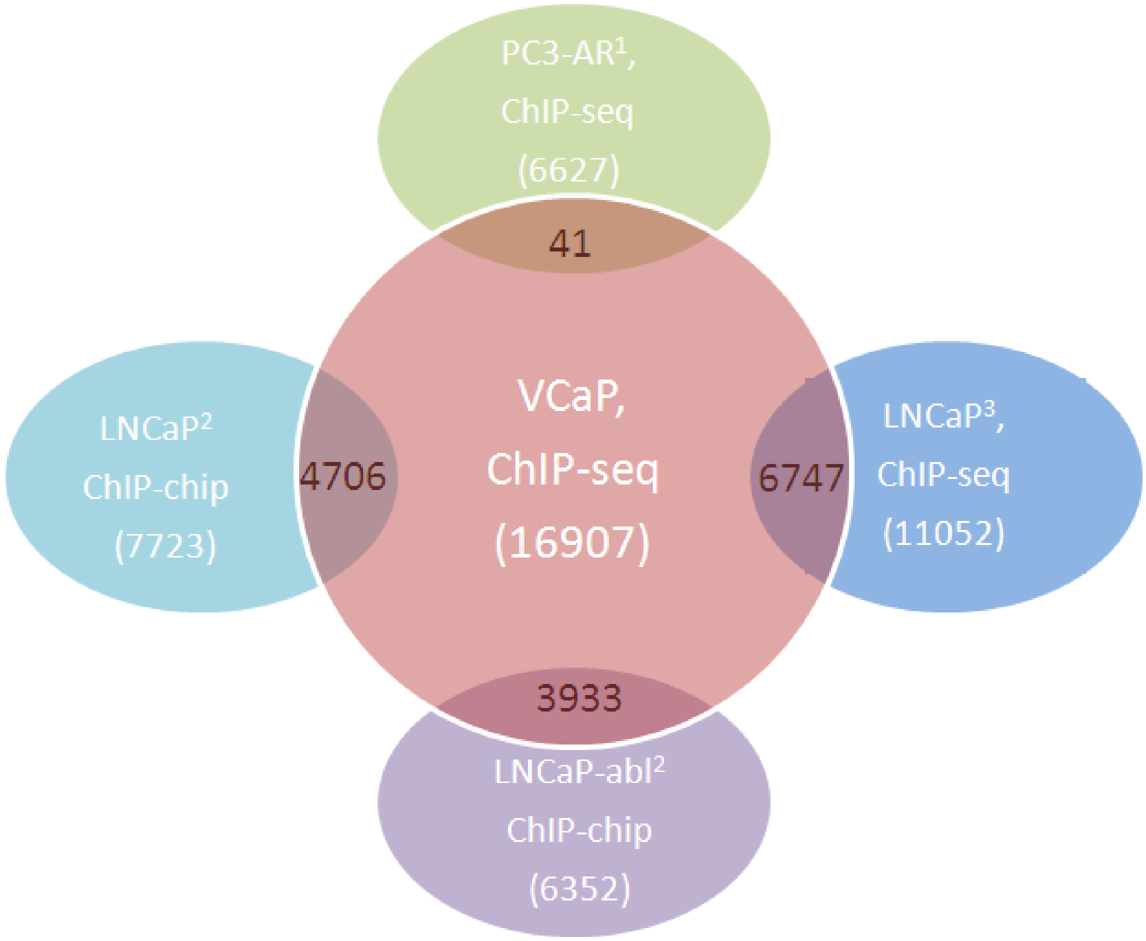

AR binding and cell type. Overlap of the AR binding sites between VCaP cells and other cell types from previous studies: [1](Lin et al., 2009); [2](Wang et al., 2009); [3](Massie et al., 2011). Binding sites based on earlier version of human genome were remapped to hg19 using UCSC liftOver tool. Overlap was defined by sharing of at least 1bp.

Figure S3

(A)

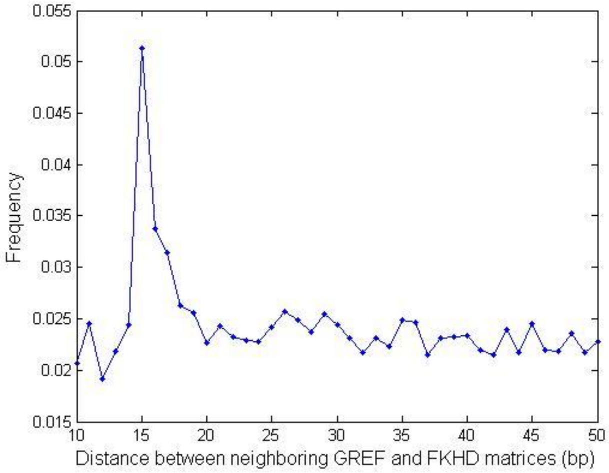

(B)

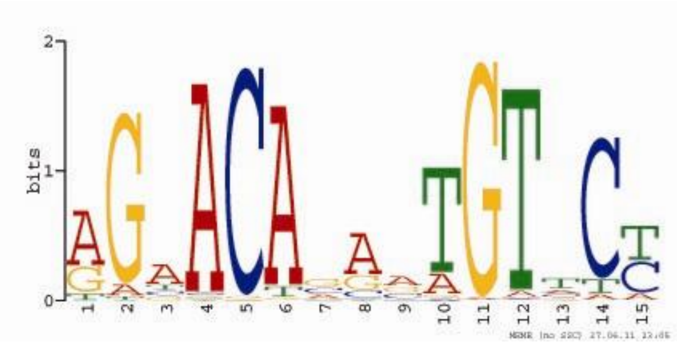

(C)

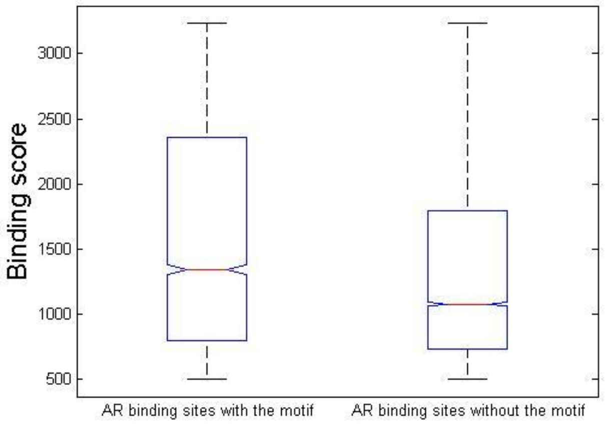

AR binding and sequence features. (A) Distance distribution between neighboring (10-50bp) GREF and FKHD elements. (B) The motif identified *de novo* from AR-bound sequences appears to be a 15bp perfect palindrome. (C) Motif and binding strength. AR binding sites were divided into two groups based on whether they had a significant occurrence of the palindromic motif in (B). Boxplots depict the distributions of their binding scores.

Figure S4

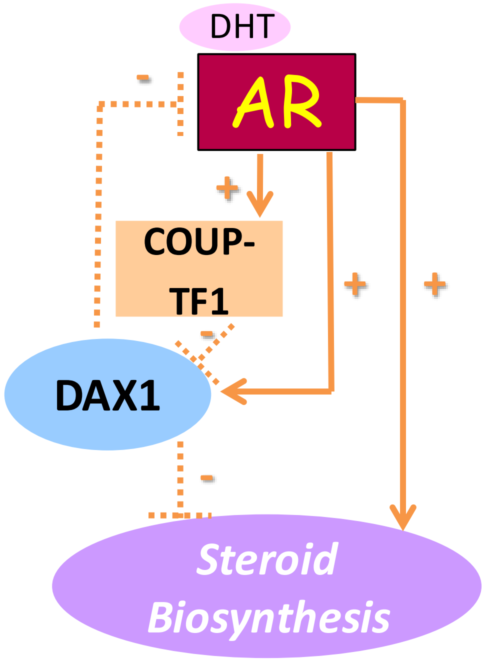

AR and DAX1 form a tightly controlled feedback loop on steroid biosynthesis: AR and DAX1 counter-balance each other's effect on steroidogenesis. AR also directly and positively regulates the expression of DAX1, whereas their physical interaction may serve to sense and prevent the over-production of DAX1 by AR. Dashed links refer to previously reported regulatory relationships, while solid links describe regulatory relationship identified in this study. Positive or stimulatory effects are represented by (+), and negative or inhibitory effects are represented by (-).
