# Supplementary material for: Dose-dependent effects of small-molecule antagonists on the genomic landscape of androgen receptor binding
Source: BMC Genomics. 2012 Jul 31;13:355. doi: 10.1186/1471-2164-13-355 (PMC3507642; doi:10.1186/1471-2164-13-355)
Supplement: Additional file 2 — sFile1. AR binding sites list. [file 1471-2164-13-355-S2.pdf]

Table S1: The distribution of AR binding sites relative to genomic annotations

	R1881(+)		R1881(-)	
**Type of genomic elements**	Percentage	Enrichment compared to genome	Percentage	Enrichment compared to genome
**Promoter**	4.1	2.2	3.7	1.9
**Exonic, complete**	1.5	0.4	1.2	0.3
**Intronic, complete**	44.5	1.1	42.2	1.0
**Intergenic**	46.7	0.8	49.2	0.9

Table S2: Gene signatures most enriched among AR-bound genes

Signature	R1881(+)	R1881(-)
ANDROGEN_RECEPTOR_NETPATH	1.75E-20	1.54E-6
PROSTATE_LNCaP_HENDRIKSEN06	4.83E-19	4.57E-6
NELSON_ANDROGEN	4.62E-12	4.43E-4
TTGTTT_V$FOXO4_01	8.76E-10	1.42E-3
V$CEBP_Q2_01	2.52E-7	8.75E-4

Statistical significance of signature enrichment was determined using cumulative hypergeometric probability distribution as previously described (Tavazoie et al., 1999).

Table S3: MatBase motifs most over-represented among AR-bound sequences, sorted by descending Z-score

	TF Family	Description	Z-Score
**R1881(+)**	V$GREF	Glucocorticoid responsive and related elements	131.43
	V$FKHD	Fork head domain factors	80.74
	V$GATA	GATA binding factors	45.3
	V$ABDB	Abdominal-B type homeodomain transcription factors	38.61
	V$HNF1	Hepatic Nuclear Factor 1	36.14
	V$NF1F	Nuclear factor 1	29.85
	V$PARF	PAR/bZIP family	29.26
	V$MYBL	Cellular and viral myb-like transcriptional regulators	28.57
	V$MYT1	MYT1 C2HC zinc finger protein	25.54
	V$CDXF	Vertebrate caudal related homeodomain protein	25.24
	V$STAT	Signal transducer and activator of transcription	23.15
	V$HOMF	Homeodomain transcription factors	22.33
	V$GRHL	Grainyhead-like transcription factors	22.32
	V$OVOL	OVO homolog-like transcription factors	20.48
	V$DMRT	DM domain-containing transcription factors	20.38
**R1881(-)**	V$GREF	Glucocorticoid responsive and related elements	41.33
	V$FKHD	Fork head domain factors	35.21
	V$ABDB	Abdominal-B type homeodomain transcription factors	20.2
	V$GATA	GATA binding factors	19.66
	V$HNF1	Hepatic Nuclear Factor 1	17.62
	V$CDXF	Vertebrate caudal related homeodomain protein	14.44
	V$PARF	PAR/bZIP family	13.75
	V$MYT1	MYT1 C2HC zinc finger protein	11.23
	V$HOMF	Homeodomain transcription factors	11.17

Over-representation statistics were reported as Z-score (the distance from the population mean in units of the population standard deviation) computed against genomic background (NCBI37/hg19).

Table S4: Transcription factor binding motifs associated with mode of AR regulation

Motif	Family	P-value[a]	% activation[b]	% repression[c]	Biased towards
THR.01	RXRF (RXR heterodimer binding sites)	0.0022	8.49	15.45	repression
ESRRB.01	EREF (Estrogen response elements)	0.0049	1.59	5.36	repression
ZFP410.01	ZF05 (C2H2 zinc finger transcription factors 5)	0.0066	8.22	3.65	activation
JUNDM2.01	CREB (cAMP-responsive element binding proteins)	0.01	0.00	1.72	repression
SIX4.01	SIXF (Sine oculis (SIX) homeodomain factors)	0.0131	13.53	8.15	activation
TEF_HLF.01	PARF (PAR/bZIP family)	0.0133	1.33	4.29	repression
GSC.02	BCDF (Bicoid-like homeodomain transcription factors)	0.0139	7.69	3.65	activation
MEF2.01	MEF2 (MEF2, myocyte-specific enhancer binding factor)	0.0213	5.84	2.58	activation
SIX6.01	SIXF (Sine oculis (SIX) homeodomain factors)	0.0234	4.51	1.72	activation
VERBA.01	RORA (v-ERB and RAR-related orphan receptor alpha)	0.0271	1.59	4.29	repression
PLAGL1.01	PLAG (Pleomorphic adenoma gene)	0.0277	6.37	10.73	repression
ESRRA.02	EREF (Estrogen response elements)	0.0282	7.43	12.02	repression
ZF5.02	ZF5F (ZF5 POZ domain zinc finger)	0.0283	3.45	1.07	activation
CREB2CJUN.01	CREB (cAMP-responsive element binding proteins)	0.034	2.39	5.36	repression
RREB1.01	RREB (Ras-responsive element binding protein)	0.0377	3.18	6.44	repression
ERR.01	EREF (Estrogen response elements)	0.0379	8.75	13.52	repression
OBOX2.01	BCDF (Bicoid-like homeodomain transcription factors)	0.0458	3.18	1.07	activation
ATF.02	CREB (cAMP-responsive element binding proteins)	0.0466	2.92	6.01	repression
DMRT1.01	DMRT (DM domain-containing transcription factors)	0.0475	0.27	1.72	repression

[a]P-value on the null hypothesis that mode of AR regulation (activation/repression) is independent of the motif (presence/absence) from two-tail Fisher's exact test

[b]Percentage of AR binding sites near (<25kb of TSS) direct activation targets with the motif

[c]Percentage of AR binding sites near (<25kb of TSS) direct repression targets with the motif

Table S5: Selective drug-modulated direct downstream effectors of AR involved in steroid metabolism

Gene	Full Name	Function	Direction
HMGCR	3-hydroxy-3-methylglutaryl-Coenzyme A reductase	Rate-controlling enzyme of the mevalonate pathway, produces cholesterol and other isoprenoids.	Direct AR-activation/ Compound 30-down regulated
STARD4	StAR-related lipid transfer (START) domain containing 4	Cholesterol homeostasis	Direct AR-activation/ Compound 30-down regulated
CYP51A1	cytochrome P450, family 51, subfamily A, polypeptide 1	Catalyzes many reactions involved in drug metabolism and synthesis of cholesterol, steroids and other lipids	Direct AR-activation/ Compound 30-down regulated
DHCR24	24-dehydrocholesterol reductase	Catalyzes the reduction of the delta-24 double bond of sterol intermediates during cholesterol biosynthesis	Direct AR-activation/ Compound 30-down regulated
SULT2B1	Sulfotransferase family, cytosolic, 2B, member 1	Involved in regulating prostate cell responsiveness to DHEA and Delta(5)-Adiol. Inhibition of SULT2B1b increased cell proliferation	Direct AR-repression/ Compound 30-up regulated
PAPSS1	3'-phosphoadenosine 5'-phosphosulfate synthase 1	An essential enzyme to synthesize activated PAPS, the sulfate donor cosubstrate for all sulfotranferases	Direct AR-repression/ Compound 30-up regulated
ACOX2	Acyl-Coenzyme A oxidase 2, branched chain	Encodes the branched-chain acyl-CoA oxidase which is involved in the degradation of long branched fatty acids and bile acid intermediates in peroxisomes. The enzyme is downregulated in CaP compared to normal	Direct AR-repression/ Compound 30-up regulated
